# Large–Small-Scale Structure Blended U-Net for Brightening Low-Light Images

**DOI:** 10.3390/s25113382

**Published:** 2025-05-28

**Authors:** Hao Cheng, Kaixin Pan, Haoxiang Lu, Wenhao Wang, Zhenbing Liu

**Affiliations:** School of Computer and Information Security, Guilin University of Electronic Technology, Guilin 541004, China; chenghao@guet.edu.cn (H.C.); 2200310220@mails.guet.edu.cn (K.P.); 19031005011@mails.guet.edu.cn (W.W.); zbliu@guet.edu.cn (Z.L.)

**Keywords:** image enhancement, color correction, large–small-scale structure, U-Net

## Abstract

Numerous existing methods demonstrate impressive performance in brightening low-illumination images but fail in detail enhancement and color correction. To tackle these challenges, this paper proposes a dual-branch network including three main parts: color space transformation, a color correction network (CC-Net), and a light-boosting network (LB-Net). Specifically, we first transfer the input into the CIELAB color space to extract luminosity and color components. Afterward, we employ LB-Net to effectively explore multiscale features via a carefully designed large–small-scale structure, which can adaptively adjust the brightness of the input images. And we use CC-Net, a U-shaped network, to generate noise-free images with vivid color. Additionally, an efficient feature interaction module is introduced for the interaction of the two branches’ information. Extensive experiments on low-light image enhancement public benchmarks demonstrate that our method outperforms state-of-the-art methods in restoring the quality of low-light images. Furthermore, experiments further indicate that our method significantly enhances performance in object detection under low-light conditions.

## 1. Introduction

Images and videos play significant roles in our daily lives, as they transmit messages and record significant moments. However, images captured under insufficient lighting conditions (e.g., nighttime) typically present color casts, as well as unnatural-looking visibility, and further deliver unsatisfactory or incorrect information for object detection and other advanced vision tasks [1,2]. Although it is possible to increase the brightness of an image by using a flashlight, increasing the ISO, or extending the exposure time during the shooting stage, these techniques require the photographer to have both advanced hardware and high technical proficiency [2,3].

Image enhancement techniques can effectively yield visually pleasing results from degraded inputs without upgrading equipment. Many traditional enhancement approaches, including pixel transformation-based [4], Retinex-based [5], and fusion-based [6,7] methods, have been reported to improve the quality of low-light images. Among them, the former directly processes the image pixels for generating high-quality images while introducing observable local over-enhancement. Retinex-based methods to enhance low-light images typically follow a “decomposition–adjustment–exposure control” process, but they yield extra color casts. Fusion-based techniques fuse multiple feature maps according to a certain rule for achieving promising performance but fail in detail enhancement.

Recently, the deep convolutional neural network (CNN) has been widely applied in medical image processing, object detection, image segmentation, etc., due to its powerful feature extraction and representation capabilities [8,9]. LLNet [10], based on the sparse denoising autoencoder, is a deep learning pioneering network which can achieve the goals of light enhancement and denoising. Subsequently, Retinex theory [11], semantic information [12], contrastive learning [13], and other technologies [14,15] were introduced into the CNN to improve its nonlinear mapping capacity from a degraded input to the corresponding high-quality image. Although these proposed learning-based methods can solve the problems encountered in traditional methods, they exhibit massive parameter space and heavily rely on computational resources. And many CNN-based models seldom utilize multiscale features at different scale spaces, introducing color distortion, blurry details, and unsatisfactory visual experiences.

**Figure 1:**
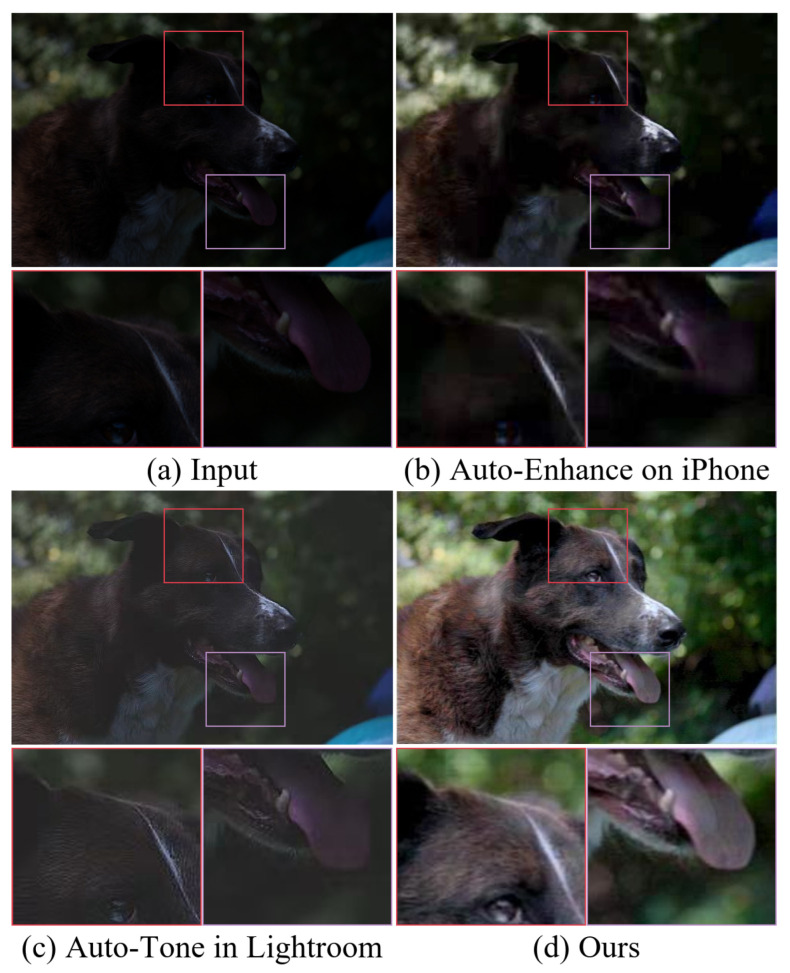
Visual comparison on low-light image. (**a**) Low-light image randomly selected from MIT-Adobe FiveK. Light-enhanced results of (**b**) iPhone, (**c**) Lightroom, and (**d**) the proposed method. Clearly, our method can effectively brighten low-light images and remove color distortion.

In this paper, we provide a feasible LLIE method called **L**arge–small-scale **S**tructure blended **U**-shaped **net**work (LSUNet). LSUNet can effectively remove color casts and noise from images captured under suboptimal lighting conditions. Specifically, the original data are transferred into the HSV color space for separating the luminosity and color components to effectively overcome color casts. A light-boosting network (LB-Net) is used for the luminosity component, i.e., channel L, for light enhancement, and a color correction network (CC-Net) is employed for the color components, i.e., channel A and channel B, for color correction. Additionally, we also design an efficient feature interaction module (EFIM) to transfer luminosity and color features between CC-Net and LB-Net. Figure 1 shows the results generated by our method and other existing techniques. Intuitively, our method produces visually satisfactory images with high contrast, vivid color, and clearer details thanks to our carefully designed modules.

To summarize, our main contributions are as follows:

- We propose a robust and feasible LLIE method which can process the luminosity and color components of low-light images in the HSV space. Experiments show that our LSUNet can yield noise-free and natural-looking images with clear details and vivid color. Additionally, our method is also helpful for object detection under low-light conditions.
- We propose a lightweight light-boosting network built on a large–small-scale structure for fully exploring multiscale features at different scale spaces. In comparison with existing multiscale structures, our method offers faster speed for light enhancement without extra observable under- and over-enhancement.
- We propose a color correction network based on a U-shaped network with a strided convolution strategy to remove color casts and noise. Additionally, an efficient feature interaction module (EFIM) is also presented to explore the relationship of luminosity and color features for generating natural-looking visibility and clearer details.

The remainder of this paper is organized as follows: In Section 2, we provide an overview of works related to the proposed approach. In Section 3, we introduce the proposed method in detail. In Section 4, we discuss the train details and benchmarks, as well as an ablation study, a comparison of computational complexity, and an application test. Finally, we present the conclusions on the proposed method in Section 5.

## 2. Related Work

Images captured under poor lighting conditions typically exhibit unsatisfactory visibility and low contrast. Consequently, plenty of LLIE techniques including traditional and learning-based methods have been proposed to improve the quality of degraded images.

### 2.1. Traditional Methods

In the early stage, pixel transformation-based and histogram equalization (HE)-based methods are usually used to restore image quality. The former, including Linear stretch, Gamma correction, and S-type function, can simplify the brightening of low-illumination images [16,17], but they introduce observable local over-/under-enhancement because of pixel stretch globally. HE-based methods, based on the statistical information of pixels including global/local histogram equalization, have been widely used for restoring the quality of degraded images acquired under suboptimal lighting conditions [18]. Huang et al. [19] proposed contrast limited dynamic quadri-histogram equalization, which includes sub-histogram partition and adaptive histogram clipping, as well as sub-histogram mapping and equalization. Dyke et al. [20] proposed an adaptive kernel-based method that seeks to address the issue of histogram sparsity for downstream applications. Yuan et al. [21] presented an adaptive histogram equalization method with visual perception consistency. Additionally, the genetic algorithm [22], multi-stage processing [23], the differential evolution algorithm [24], and other techniques have been proposed to optimize HE-based methods. But these methods show poor performance in robustness and generalization for the LLIE task and inevitability introduce unwanted color casts and blurry details.

Physical model-based methods, including Retinex and the atmospheric scattering model, exhibit good interpretability in light enhancement. Retinex-based methods [25,26] separate the image into illumination and reflectance components to brighten low-illumination images. Cai et al. [27] employed a cortex-like contour extraction algorithm and retina-inspired textural gradient detection for Retinex decomposition. Jia et al. [28] proposed a robust Retinex-based model with reflectance map re-weighting to improve and re-balance brightness. Veluchamy et al. [29] proposed a Retinex variational decomposition-based detail-preserving noise suppression model to address quality degradation issues in low-light images. Yang et al. [30] proposed the Weighted Low-Rank Tensor regularization Retinex (WLRT-Retinex) model, which introduces weighted low-rank tensor priors in the Retinex decomposition process. These Retinex-based methods suffer from observable color deviation and blurry details. Jeon et al. [31] designed an efficient and fast low-light image enhancement method using an atmospheric scattering model based on an inverted low-light image. Zhang et al. [32] utilized the atmospheric scattering model and color correction to yield a dehazed background sub-image.

### 2.2. Learning-Based Methods

Deep learning has revolutionized low-light enhancement, with convolutional neural network (CNN)-based methods [33,34] leading recent advancements. Wei et al. [35] incorporated Retinex theory into CNNs and proposed deep Retinex-Net for illumination adjustment. Zhang et al. [36] further built an effective network, Kindling the Darkness. Zhu et al. [37] proposed a robust Retinex decomposition network (named RRDNet) to predict noise for denoising while restoring underexposed images. Jiang et al. [38] reported a Retinex-based real-low to real-normal network which employs Decom-Net, Denoise-Net, and Relight-Net for decomposing, denoising, and performing contrast enhancement, respectively. Wu et al. [39] proposed a category-specific processing network which crops an input into patches and classifies these patches into “simple”, “medium”, and “hard” categories based on their information. Lim et al. [40] designed a deep stacked Laplacian restorer (DSLR) by leveraging useful properties of the Laplacian pyramid both in image and feature spaces. Notably, Zero-DCE [41] achieves unsupervised enhancement through learnable nonlinear curves, which are further optimized in Zero-DCE++ [42] by using lightweight depth-wise convolutions. Self-supervised approaches, such as maximum-entropy Retinex [43] and EnlightenGAN [14], reduce reliance on paired data.

Recently, researchers introduced Transformers from the field of NLP into low-light image enhancement, such as Retinexformer [44], a one-stage Retinex-based low-light enhancement framework. Xu et al. [45] proposed an unbalanced point-guided multiscale Transformer-based conditional normalizing flow for low-light image enhancement. Kou et al. [46] proposed a lightweight two-stage Transformer which contains an FFT-guidance block (FGB) and YOLOv3. Jiang et al. [47] proposed a Retinex-based framework to characterize the specific knowledge of the reflectance and illumination components while removing perturbation. Wang et al. [48] designed a luminance and chrominance dual-branch network, termed LCDBNet, for low-light image enhancement which divides low-light image enhancement into two sub-tasks, i.e., luminance adjustment and chrominance restoration. In addition, Nguyen et al. [49] proposed an LLIE method using a conditional diffusion model, which can effectively reduce training time and computational cost. Jiang et al. [50] proposed a diffusion-based unsupervised framework that incorporates physically explainable Retinex theory with diffusion models for low-light image enhancement. Although these learning-based methods show powerful generalization and robustness in the LLIE task, they yield color casts in the light-enhanced results.

## 3. Method

### 3.1. Motivation

Low-light images present low brightness and contrast, as well as unsatisfactory visibility. Although numerous learning-based methods show better performance in light enhancement and contrast stretch in the LLIE task in the RGB color space, they inevitably yield color casts and blurry details in the light-enhanced results. The main reason may be that the R, G, and B channels exhibit a strong correlation in color representation. Previous studies, e.g., Zero DCE [41] and CIDNet [51], have proven that transferring the image captured under suboptimal lighting conditions into the HSV, LAB, or other color spaces to separate the luminosity and color components can remove color casts to some extent. However, these methods present either poor multiscale feature representation or high computational complexity.

Inspired by Zero DCE [41], we design a large–small-scale structure blended U-shaped network to enhance the quality of low-light images in the LAB color space. Specifically, a large–small-scale structure and an enhanced U-shaped network are carefully designed to explore multiscale hierarchical features. And we also design an efficient feature interaction module (EFIM) to explore the relationship of luminosity and color features.

### 3.2. Network Architecture

**Figure 2:**
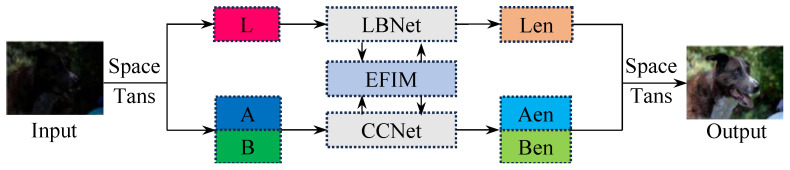
The architecture of the proposed method (LSUNet), which contains a light-boosting network (LB-Net), a color correction network (CC-Net), and an efficient feature interaction module (EFIM). The LB-Net is built upon the enhanced U-shaped network, the CC-Net is composed of the large–small-scale structure, and the EFIM is designed based on collaborative cross attention.

As shown in Figure 2, the proposed network, LSUNet, consists of a light-boosting network (LB-Net), a color correction network (CC-Net), and an efficient feature interaction module (EFIM).

The input low-light image $X_{in}$ is first transferred into the LAB color space to separate its luminosity feature $X_{L}$ and color feature $X_{A,B}$, a stage that can be formulated as

(1) $$X_{L},X_{A,B}=\mathrm{Space}\_\mathrm{trans}\left(X_{in}\right)$$

where $\mathrm{Space}\_\mathrm{trans}\left(\cdot \right)$ denotes the color space transformation operation.

Subsequently, we apply the LB-Net and the CC-Net on luminosity feature $X_{L}$ and color feature $X_{A,B}$ to perform light boosting and color correction, respectively, for images acquired under suboptimal lighting conditions.

(2) $$\left\{\begin{array}{c} L_{en}=\mathrm{LBNet}\left(X_{L}\right)\\ A_{en},B_{en}=\mathrm{CCNet}\left(X_{A,B}\right) \end{array}\right.$$

where $L_{en}$, $A_{en}$, and $B_{en}$ denote the enhanced luminosity and color features, respectively. $\mathrm{LBNet}\left(\cdot \right)$ and $\mathrm{CCNet}\left(\cdot \right)$ stand for the light-boosting and color correction operations. Additionally, the EFIM is employed for feature interaction in both branches.

#### 3.2.1. Color Space Transformation

In the RGB color space, the image’s A, B, and C channels exhibit strong relationships in color representation. Hence, LLIE methods can restore the quality of low-light images in the RGB color space, but they may introduce observable color casts and cause an unnatural appearance. To handle this problem, we process the luminosity and color components of low-light images in the LAB color space for yielding visually pleasing images with vivid color. Specifically, we first normalize the pixels of the input image to $\left[0,1\right]$; then, the R, G, and B channels of the normalized pixels are processed by the linear transformation matrix $M_{\mathrm{RGB}2\mathrm{XYZ}}$ to obtain X, Y, and Z features. This can be defined as

(3) $$\left[\begin{array}{ccc} X & Y & Z \end{array}\right]=\left[\begin{array}{ccc} R & G & B \end{array}\right]M_{\mathrm{RGB}2\mathrm{XYZ}}$$

So, the image pixels values in the LAB color space can be calculated as

(4) $$\left\{\begin{array}{c} L=116\cdot f\left(Y\right)-16\\ A=500\cdot \left(f\left(X\right)-f\left(Y\right)\right)\\ B=500\cdot \left(f\left(Y\right)-f\left(Z\right)\right) \end{array}\right.$$

where $f\left(\cdot \right)$ is the nonlinear transformation function, which can be expressed as

(5) ft=t13,ift≥619313·2962·t+16116,else

where $t\in \left\{X,Y,Z\right\}$, and the inverse transformation can be defined as

(6) $$\left[\begin{array}{ccc} R & G & B \end{array}\right]=\left[\begin{array}{ccc} X & Y & Z \end{array}\right]{\left(M_{\mathrm{RGB}2\mathrm{XYZ}}\right)}^{-1}$$

where ${\left(M_{\mathrm{RGB}2\mathrm{XYZ}}\right)}^{-1}$ denotes the inverse of the linear transformation matrix $M_{\mathrm{RGB}2\mathrm{XYZ}}$.

#### 3.2.2. Color Correction Network

**Figure 3:**
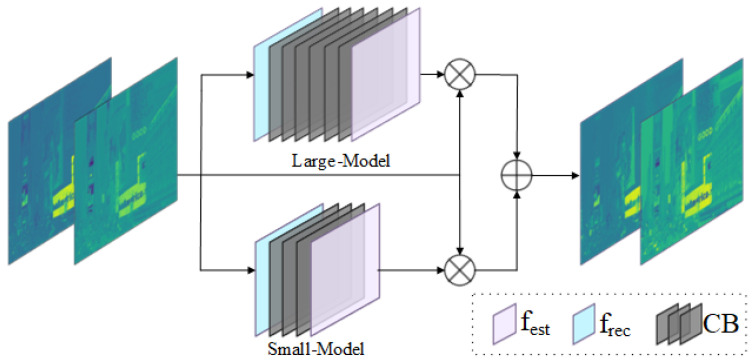
The structure of our CC-Net, which consists of a small-scale module (SM) and a large-scale model (LM). Notably, these modules include a feature extraction layer, a convolution block, and a feature reconstruction layer.

Numerous LLIE methods cannot successfully remove color casts. Additionally, image features exhibit different representations at different scale spaces. Therefore, we propose a color correction network (CC-Net), a dual-branch structure including a large-scale module (LM) and a small-scale module (SM), to explore the multiscale features of the A and B channels. Notably, these two modules consist of a feature extraction layer $f_{est}$, a convolution block (CB), and a feature reconstruction layer $f_{rec}$. The detailed architecture is shown in Figure 3 and Table 1. In this work, we apply the CC-Net on the A and B channels to avoid color casts for low-light images.

Specifically, we feed the color components, i.e., the A and B channels, into CC-Net. In the LM, the feature extraction layer $f_{est}$ is utilized to extract the shallow features. Then, the convolution block (CB), consisting of 3×3 Conv and 1×1 Conv, is applied to explore the depth feature. Finally, a feature reconstruction layer $f_{rec}$ is used to refine the depth feature and further multiply it by the input in a pixel-by-pixel manner to obtain the large-scale feature $F_{L}$. This stage can be formulated as

(7) $$F_{L}=f_{rec}^{L}\left(H_{CB}^{L}\left(f_{est}^{L}\left(X_{A,B}\right)\right)\right)⊗X_{A,B}$$

where ⊗ denotes pixel-wise multiplication and $H_{CB}^{L}\left(\cdot \right)$ denotes the convolution block (CB) in the LM. $X_{A,B}$ denotes the A and B channels of the input. The SM presents similar processing to the LM, but its CB contains a different number of convolutions. The small-scale feature $F_{S}$ can be defined as

(8) $$F_{S}=f_{rec}^{S}\left(H_{CB}^{S}\left(f_{est}^{S}\left(X_{A,B}\right)\right)\right)⊗X_{A,B}$$

Finally, we integrate the small-scale feature $F_{S}$ and the large-scale feature $F_{L}$ for generating enhanced A and B channels as

(9) $${\hat{X}}_{A,B}=F_{L}⊕F_{S}$$

where ${\hat{X}}_{A,B}$ denotes the enhanced A and B channels and ⊕ denotes pixel-wise summation. Through the above processing, we can effectively mine multiscale features to achieve the goals of color correction and detail enhancement in low-light image enhancement.

#### 3.2.3. Light-Boosting Network

**Figure 4:**
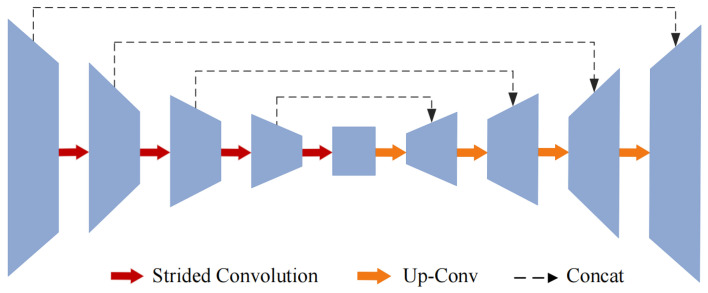
The structure of LB-Net including an encoder, a decoder, and a strided convolution.

In our work, we propose a light-boosting network (LB-Net) based on the U-shaped network to process the luminosity component for light enhancement. The structure of LB-Net is shown in Figure 4 and consists of an encoder, a decoder, and a skip connection. The encoder employs four convolutions with batch normalization and ReLU function (CBRs) for detecting multi-level features. In addition, we integrate a strided convolution into two successive CBRs rather than max pooling to reduce computational complexity. Given the luminosity component $X_{L}$ of an input image, the encoding stage $F_{enc}$ can be defined as

(10) $$\left\{\begin{array}{c} CBR_{n}^{i}=ReLU\left(BN\left(Conv_{n}\left(X_{L}\right)\right)\right)\\ F_{enc}=f_{sc}\left({CBR}_{n↓}^{i}\right)\to CBR_{n}^{i+1} \end{array}\right.$$

where $Conv_{n}\left(\cdot \right)$ denotes an n×n convolution operation, $BN\left(\cdot \right)$ denotes batch normalization, $ReLU\left(\cdot \right)$ denotes the rectified linear unit function, $f_{sc}\left(\cdot \right)$ denotes the strided convolution, ↓ denotes downsampling, and → indicates that the feature generated by the $i_{th}$ CBR is fed into the ${i+1}_{th}$ one.

The decoder has the same structure as the encoder, and the encoded feature $F_{enc}\in R^{\frac{h}{2}\times \frac{w}{2}\times 2c}$ is processed by the successive upsampling operation and the CBR to restore the feature maps of the encoder to their original resolution. Additionally, we also employ the skip connection between the $i_{th}$ CBR in the encoder and its corresponding CBR in the decoder to fully explore the hierarchical features. Finally, the output $F_{out}\in R^{\frac{h}{16}\times \frac{w}{16}\times 16c}$ can be defined as

(11) $$F_{out}=\mathrm{concat}\left(CBR^{i},{\left[CBR^{l}\right]}_{↑}\right)\to CBR^{l+1}$$

where *i* is chosen from the largest to the smallest in $\left\{1,2,3,4\right\}$ and *l* is chosen in the opposite order. $\mathrm{concat}\left(\cdot \right)$ denotes the concatenation operation. ↑ denotes the upsampling operation.

#### 3.2.4. Efficient Feature Interaction Module

**Figure 5:**
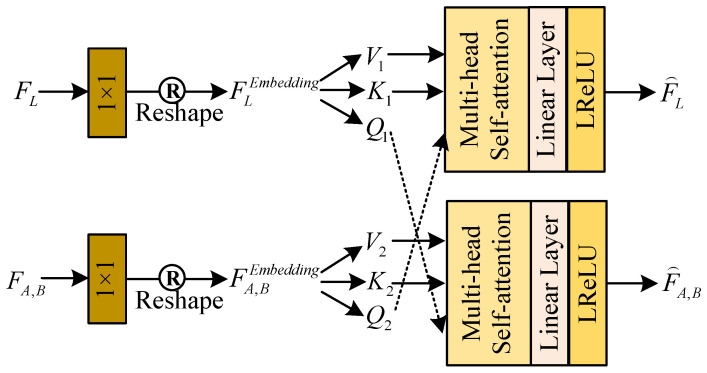
The structure of the EFIM.

For taking full advantage of the correlation and complementarity of luminosity and color features, we design an efficient feature interaction module (EFIM) for information interaction between LB-Net and CC-Net. And the structure of the proposed EFIM is shown in Figure 5.

Firstly, we employ a Conv layer with kernel sizes of 1×1 and the reshape operation on $F_{L}$ and $F_{A,B}$ to generate new sequence features $F_{L}^{Embedding}$ and $F_{A,B}^{Embedding}$. Then, they are further split into query (Q), key (K), and value (V) subsequences with the same dimension.

(12) $$\left\{\begin{array}{c} Q_{1},K_{1},V_{1}=\mathrm{Chunk}\left(\mathrm{Reshape}\left(f_{1\times 1}\left(F_{L}\right)\right)\right)\\ Q_{2},K_{2},V_{2}=\mathrm{Chunk}\left(\mathrm{Reshape}\left(f_{1\times 1}\left(F_{A,B}\right)\right)\right) \end{array}\right.$$

where $\mathrm{Chunk}\left(\cdot \right)$ denotes the split operation, $\mathrm{Reshape}\left(\cdot \right)$ denotes the feature reshaping operation, and $f_{1\times 1}()$ denotes the Conv layer with a kernel size of 1×1.

Subsequently, we exchange $Q_{1}$ and $Q_{2}$, further perform successive multi-head self-attention, and apply a linear layer and the LeakyReLU function on $\left(Q_{2},K_{1},V_{1}\right)$ and $\left(Q_{1},K_{2},V_{2}\right)$ to generate mixed features ${\overset{⌢}{\;F}}_{L}$ and ${\overset{⌢}{\;F}}_{A,B}$ They can be defined as

(13) $$\left\{\begin{array}{c} {\overset{⌢}{\;F}}_{L}=\mathrm{LReLU}\left(\mathrm{Linear}\left(\mathrm{MultiHead}\left(Q_{2},K_{1},V\right)\right)\right)\\ {\overset{⌢}{\;F}}_{A,B}=\mathrm{LReLU}\left(\mathrm{Linear}\left(\mathrm{MultiHead}\left(Q_{1},K_{2},V_{2}\right)\right)\right) \end{array}\right.$$

### 3.3. Loss Function

In this paper, we employ the $L_{1}$, $L_{spa}$, and $L_{str}$ loss functions to train the proposed method, which can assess the difference between the enhanced image and its corresponding ground truth. We analyze these three loss functions as follows.

**$L_{1}$ Loss** can improve the results of the proposed method by minimizing the differences between the predicted value and its ground truth. It can be defined as

(14) $$L_{1}\left(X,Y\right)=\frac{1}{N}\sum_{i,j\in I}\left|X_{i}-Y_{j}\right|\;,$$

where *N* is the number of samples, *X* is the input image, and *Y* is the ground truth.

**Spatial Consistency Loss $L_{spa}$** ensures that the output and input images share similar spatial consistency by calculating the difference between adjacent pixels. $L_{spa}$ is defined as

(15) $$L_{spa}\left(X,Y\right)=\frac{2}{M}\sum_{i=1}^{M}\sum_{j\in \Omega (i)}^{M}\left(\right|Y_{i}-Y_{j}\left|-\right|X_{i}-X_{j}{\left|\right)}^{2}\;,$$

where *M* is the number of local regions and Ω(i) is the four neighboring regions (top, down, left, and right) centered at area *i*.

**Structure Similarity Loss $L_{str}$**. Compared with the $L_{1}$ and $L_{spa}$ loss functions, $L_{str}$, based on the Human Visual System (HVS), is sensitive to local structural changes in an image. Therefore, it was employed to measure the structural similarity between the enhanced image *x* and its corresponding ground truth *y*, and $L_{str}$ is defined as

(16) $$L_{str}\left(x,y\right)=1-\frac{\left(2\mu_{x}\mu_{y}+C_{1}\right)\left(\sigma_{xy}+C_{2}\right)}{\left(\mu_{x}^{2}+\mu_{y}^{2}+C_{1}\right)\left(\sigma_{x}^{2}+\sigma_{y}^{2}+C_{2}\right)},$$

where $\mu_{x}$ and $\mu_{y}$ are the pixel average value of the enhanced images and the ground truth. $\sigma_{x}^{2}$ and $\sigma_{y}^{2}$ represent the variance values of the pixels in the *x* and *y* directions, respectively; $\sigma_{xy}$ is the pixel covariance; and $C_{1}$ and $C_{2}$ are constants.

**Total Loss.** For generating visually pleasing results by the proposed method, we employ the total loss function $L_{Total}$ including three types of losses, i.e., $L_{1}$, $L_{spa}$, and $L_{str}$, to train it. $L_{Total}$ can be expressed as

(17) $$L_{Total}=\lambda_{1}L_{1}^{i}+\left(1-\lambda_{1}\right)L_{spa}^{i}+\lambda_{2}L_{str}^{i}\;,$$

where $\lambda_{1}$ and $\lambda_{2}$ represent the corresponding balance coefficients. We empirically set $\lambda_{1}=0.8$ and $\lambda_{2}=0.1$ to make our approach works well in the LLIE task.

## 4. Experiment and Analysis

In this section, we briefly describe the experimental details, datasets, and evaluation metrics. We then validate the effectiveness of the proposed method by using several public datasets. Finally, an ablation study and object detection in the dark are implemented.

### 4.1. Experimental Details

#### 4.1.1. Training Details

As illustrated in Figure 2, the original data are converted into LAB images, and the output is transformed back into the original format. We first resize all input images to dimensions of 384×384×3 before training. The Adam optimizer [52] is employed to train the proposed network, with the batch size being set to 8. The initial learning rate is established as 0.001 and is subsequently reduced by a factor of one-tenth after 50 epochs, for a total of 100 epochs. The proposed method is implemented by using the PyTorch-1.3 framework, and all validation experiments are conducted on an NVIDIA Tesla P100 GPU.

#### 4.1.2. Datasets and Evaluation Metrics

We train our method on the MIT-Adobe FiveK [53] and LOL datasets [35]. The MIT-Adobe FiveK dataset comprises 4500 training image pairs and 500 test image pairs, while the LOL dataset consists of 485 training pairs and 15 test pairs. We evaluate performance by using PSNR, SSIM, and learned perceptual image patch similarity (LPIPS) [54]. Additionally, we perform tests on real-world datasets, including MEF [55], Fusion [56], and VV, using the natural image quality evaluator (NIQE) [57], neural feature-based image quality assessment (NFERM), lightness order error (LOE) [58], and information entropy (IE) to objectively evaluate image quality.

### 4.2. Ablation Study

For better understanding LSUNet, we conduct an ablation study, including color space transformation and the use of the loss function and CC-Net, on public datasets.

#### 4.2.1. Study of Color Space Transformation

To verify the effectiveness of color space transformation, we apply the proposed network with identical configurations to improve low-illumination images in the RGB, HSV, YCbCr, Eigencolor, and LAB color spaces.

**Figure 6:**
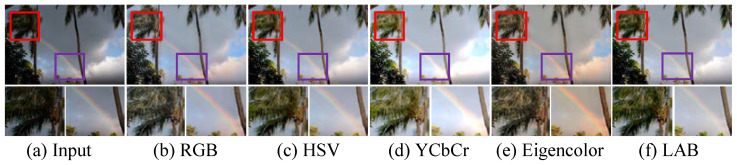
Ablation study of color space transformation. (**a**) Input image. Results generated in (**b**) RGB, (**c**) HSV, (**d**) YCbCr, (**e**) Eigencolor, and (**f**) LAB color spaces. The red and purple boxes indicate the magnified local detail regions. The red box highlights the improvement in detail clarity after enhancement, while the purple box emphasizes the accuracy and naturalness of color restoration.

Concretely, the input is first converted into the HSV or LAB color space to separate color and luminance components; then, they are fed into LB-Net and CC-Net to restore the quality of the low-light images. Figure 6 presents light-enhanced images randomly selected from the MIT-Adobe FiveK dataset in different color spaces. Illustratively, the result generated in the YCbCr color space exhibits local over-enhancement, and that generated in Eigencolor exhibits color casts, while those generated in the RGB and HSV color spaces exhibit low contrast and blurry details. In contrast, the images improved in the LAB color space exhibit natural-looking visibility, vivid color, and clearer details.

Additionally, Table 2 depicts the average PSNR, SSIM, and AG scores of our LSUNet on MIT-Adobe FiveK in different color spaces. It can be seen that LSUNet in the LAB color space exhibits comparable and higher values of the AG, PSNR, and SSIM metrics compared with the other color spaces. In summary, the qualitative and quantitative evaluations show that our method in the LAB color space has superior performance on the LLIE task.

#### 4.2.2. Study of CC-Net

To evaluate the performance of the small-scale module (SM) and the large-scale module (LM) in CC-Net, we remove them from LSUNet. Table 3 demonstrates the average PSNR and SSIM scores of these operations. The results shows that the LM outperforms the SM in PSNR and SSIM. And our CC-Net can yield comparable and satisfactory PSNR and SSIM scores, benefiting from our carefully designed SM and LM.

#### 4.2.3. Study of Loss Function

To evaluate the effectiveness of all mentioned loss functions, we train many versions of our method by using various combinations of loss functions. The average PSNR and SSIM scores on the MIT-Adobe Five-K dataset processed by these different versions of our method are presented in Table 4. As observed, $L_{1}$, $L_{spa}$, and $L_{SSIM}$ demonstrate similar performance in generating PSNR scores. In terms of SSIM values, the results indicate that $L_{SSIM}$ outperforms $L_{1}$ by 0.1508 and $L_{spa}$ by 0.0729. Overall, the total loss function effectively combines the advantages of these three losses and outperforms other combinations in generating satisfactory and comparable average PSNR and SSIM scores.

### 4.3. Benchmark Evaluations

#### 4.3.1. Comprehensive Evaluation on Synthetic Datasets

We first conduct visual comparisons and quantitative analyses on two synthetic benchmark datasets (i.e., MIT-Adobe FiveK and LOL) to demonstrate the effectiveness of our LSUNet in brightening low-light images.

**Figure 7:**
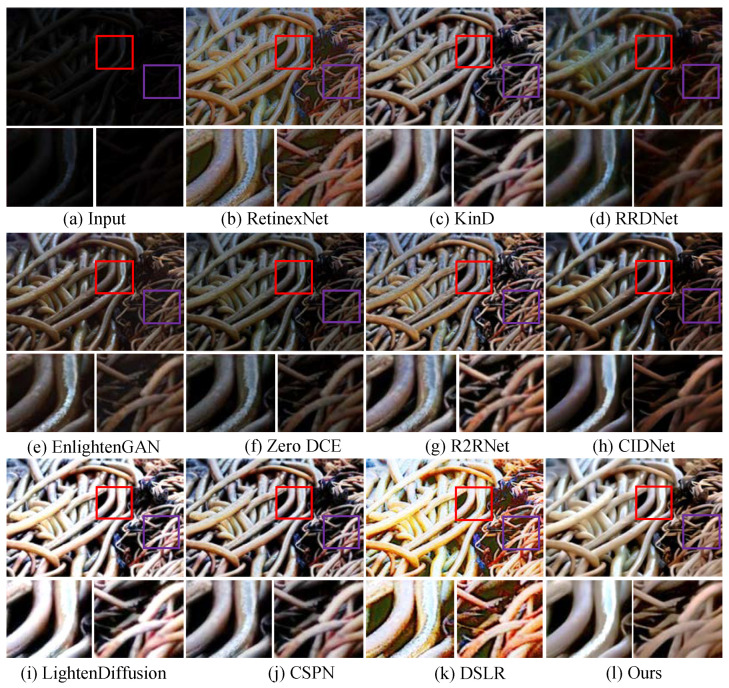
Visual comparisons of different LLIE methods on MIT-Adobe FiveK dataset. The red and purple boxes highlight local detail zoom-in regions.

**Qualitative analysis.** We first test LSUNet and other state-of-the-art LLIE methods on the MIT Adobe FiveK benchmark, and randomly selected visual comparison results are depicted in Figure 7. It can be seen that RRDNet and Zero DCE fail to increase the brightness of low-light images and make the details clearer. EnlightGAN cannot effectively tackle local darkness. RetinexNet introduces unnatural-looking visibility, blurry details, and unwanted halo artifacts in the light-enhanced results. R2RNet improves brightness, contrast, and saturation, but the light-enhanced result presents unsatisfactory structural detail. KinD shows unsatisfactory performance in detail enhancement and removing local darkness. CIDNet fails to brighten low-illumination images. DSLR significantly enhances image brightness, but it yields observable color casts and local over-enhancement. LightenDiffusion effectively improves image contrast while generating blurry details. The CSPN-enhanced image exhibits unsatisfactory contrast. In contrast, our method outperforms other comparison LLIE methods in terms of contrast stretching, detail enhancement, and color correction.

**Figure 8:**
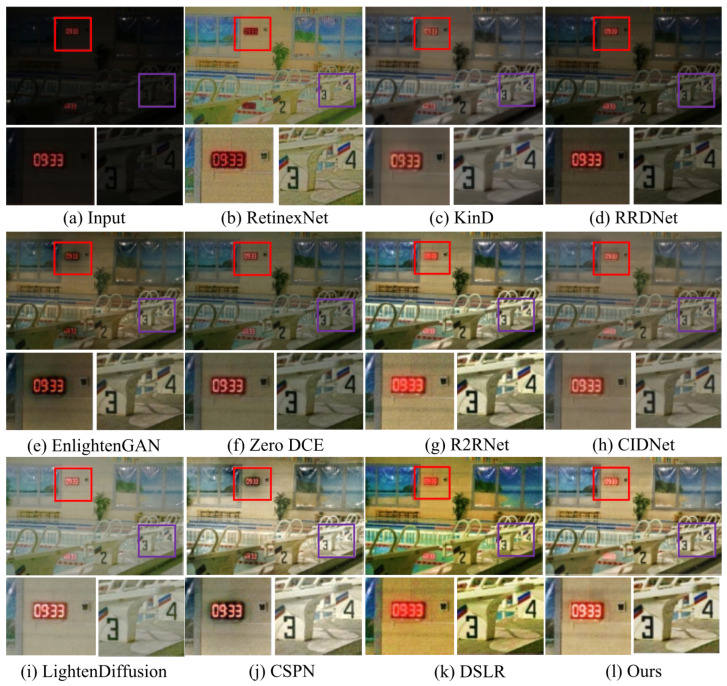
Visual comparisons of different LLIE methods on LOL dataset. The red and purple boxes highlight local detail zoom-in regions.

Additionally, Figure 8 shows the enhanced images generated by different LLIE methods. It can be easily observed that R2RNet shows satisfactory performance in light enhancement and contrast stretching while introducing observable halo artifacts and blurry details. RetinexNet cannot effectively remove inherent noise and unnatural visual appearance. RRDNet fails to yield high-quality results from the corresponding original input. EnlightenGAN and Zero DCE show similar performance on LLIE tasks, and their enhanced images still exhibit unwanted noise, local darkness, and blurry details. KinD shows satisfactory performance in detail sharpening, but it cannot remove the haze-like appearance completely and generate visually pleasing brightness. LightenDiffusion and CSPN show satisfactory performance in light enhancement, but the former generates a whitish tone in the light-enhanced images, and the latter generates unwanted halo artifacts. DSLR introduces an unnatural appearance, color casts, and amplified noise in the light-enhanced images. CIDNet shows poor performance in contrast stretching. On the contrary, LSUNet effectively removes color casts and improves visibility without observable amplified noise, over-/under-enhancement, and local darkness.

**Quantitative analysis.** To quantitatively evaluate the performance of our LSUNet, we compare it with other LLIE methods by using PSNR, SSIM, and LPIPS. The average PSNR, SSIM, and LPIPS scores of different LLIE methods on synthetic datasets including MIT-Adobe FiveK and LOL are presented in Table 5. It can be easily seen that LSUNet shows better performance in creating the highest PSNR score and the lowest LPIPS score on MIT-Adobe FiveK than the comparison methods. In addition, our method has the highest SSIM score and the lowest LPIPS score on the LOL dataset. The qualitative and quantitative analyses suggest that our carefully designed LSUNet generally yields satisfactory visibility and a natural-looking appearance in the LLIE task.

#### 4.3.2. Comprehensive Evaluation on Real Datasets

To further assess the generalization and robustness of LSUNet, we also conduct evaluation experiments on real datasets, including MEF, Fusion, and VV. Subsequently, we perform qualitative and quantitative evaluations on the light-enhanced results.

**Figure 9:**
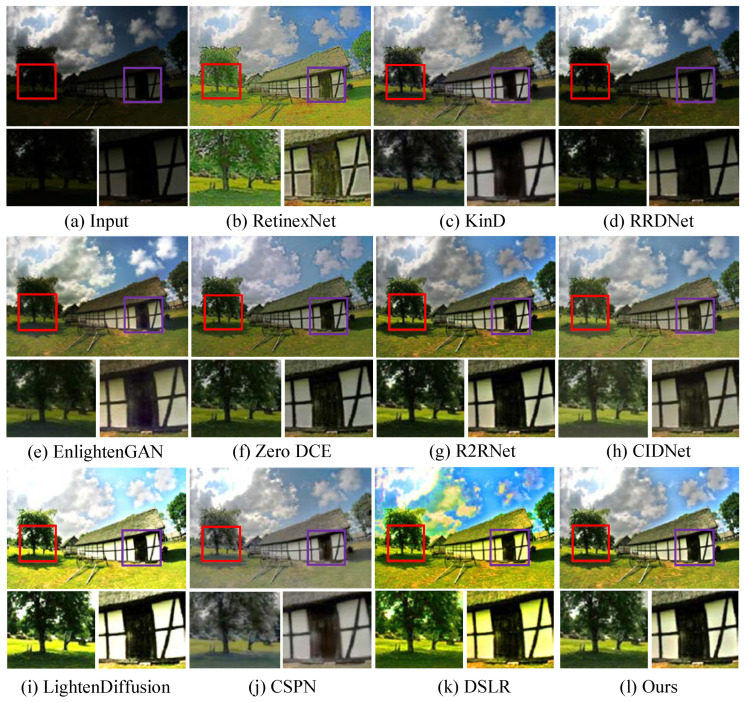
Visual comparisons of different LLIE methods on MEF dataset. The red and purple boxes highlight local detail zoom-in regions.

**Qualitative analysis.**Figure 9 shows the light-enhanced results randomly selected from the MEF dataset to test the performance of LSUNet and other comparison methods on local extremely low-light images. It can be easily found that KinD and RRDNet show poor performance in removing local darkness and contrast stretching. Although EnlightGAN, Zero DCE, and R2RNet outperform KinD and RRDNet in light enhancement for images captured under suboptimal lighting conditions, R2RNet introduces undesired halo artifacts and blurry details, Zero DCE yields a haze-like appearance in some light-enhanced images, and EnlightenGAN generates extra yellowish artifacts. RetinexNet significantly brightens low-illumination images, but the enhanced images exhibit unnatural-looking visibility and amplified noise. DSLR generates obvious halo artifacts and unnatural-looking appearance, LightenDiffusion fails in local enhancement and presents blurry details, and CSPN yields color casts. In comparison, the proposed method effectively removes local extremely low light and improves contrast without under-/over-enhancement.

**Figure 10:**
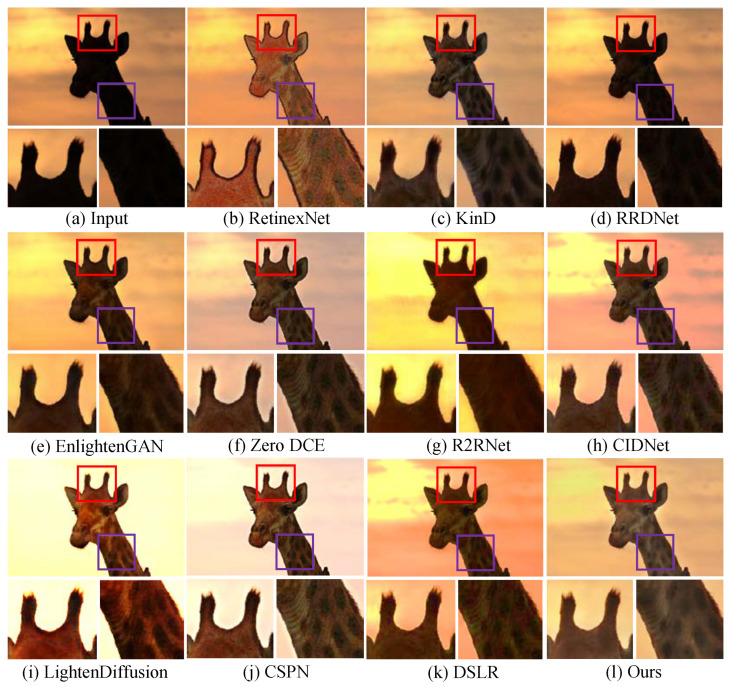
Visual comparisons of different LLIE methods on Fusion dataset. The red and purple boxes highlight local detail zoom-in regions.

**Figure 11:**
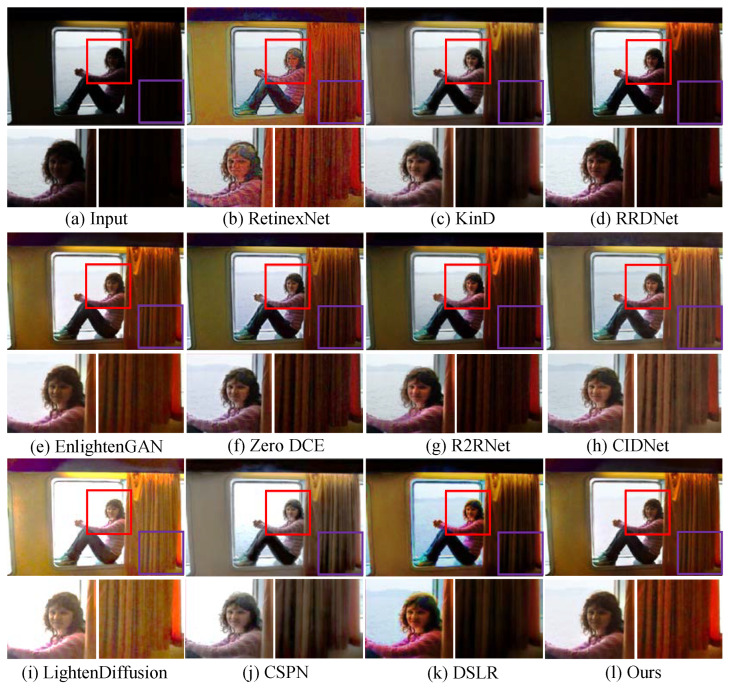
Visual comparisons of different LLIE methods on VV dataset. The red and purple boxes highlight local detail zoom-in regions.

We further test our method on the Fusion and VV public datasets, and the light-enhanced example images generated by different LLIE methods are shown in Figure 10 and Figure 11, respectively. As shown in Figure 10, Zero DCE succeeds in light enhancement in low-illumination images but introduces an observable whitish tone and blurry details. R2RNet and RRDNet fail in detail boosting and local light enhancement in a yellow low-light image. Additionally, R2RNet generates observable local over-enhancement. KinD and EnlightenGAN cannot successfully tackle low contrast, and the former yields obvious color casts. RetinexNet fails to create visually pleasing images and remove the unnatural appearance. CIDNet and DSLR cannot remove local over-enhancement and local low contrast. LightenDiffusion generates an unnatural appearance and color casts. As illustrated in Figure 11, RRDNet, KinD, and R2RNet have unsatisfactory performance in removing local extremely low-illumination areas. RetinexNet introduces obvious noise, and EnlightenGAN produces local over-enhancement. LightenDiffusion shows poor performance in color correction and denoising. DSLR fails to brighten partial darkness, and CSPN generates a whitish tone and color casts in the light-enhanced results. CIDNet is also unsuccessful in removing color casts. In contrast, our method outperforms other comparison methods in generating noise-free images with vivid color and clearer details.

**Quantitative analysis.** We perform the quantitative analysis on three public benchmarks, i.e., MEF, Fusion, and VV, to verify the performance of the different methods. The latter’s average NIQE, NFERM, IE, and LOE scores on MEF, Fusion, and VV are shown in Table 6. From the quantitative evaluation scores illustrated in Table 6, it can be observed that LSUNet can generate comparable and more satisfactory values of the NIQE, NFERM, IE, and NIQE metrics than the comparison state-of-the-art LLIE methods. Overall, our method performs satisfactorily on images acquired under low-light conditions in terms of qualitative and quantitative evaluations.

Besides the above quantitative and qualitative analyses, we further conducted subjective visual evaluation involving 30 volunteers from various age groups and professions on the MEF, Fusion, and VV public datasets. Participants blindly rated light-enhanced real-world images on a scale of 1 to 5, with higher scores indicating more satisfactory visual quality. As shown in Table 7, it can be easily found that images improved by our method exhibit the highest visual scores. That is, our method outperforms other state-of-the-art LLIE methods in yielding natural-looking images with satisfactory visibility.

### 4.4. Comprehensive Evaluation of Computational Complexity

We further compare computational complexity, including Param, Flops, and runtime, of all the above-listed LLIE methods on the LOL dataset to verify the efficiency of LSUNet, and the results are illustrated in Table 8. It can be easily found that our method is superior to other comparison methods in computational complexity. That is to say, our method can effectively restore the quality of low-illumination images.

### 4.5. Object Detection in the Dark

**Figure 12:**
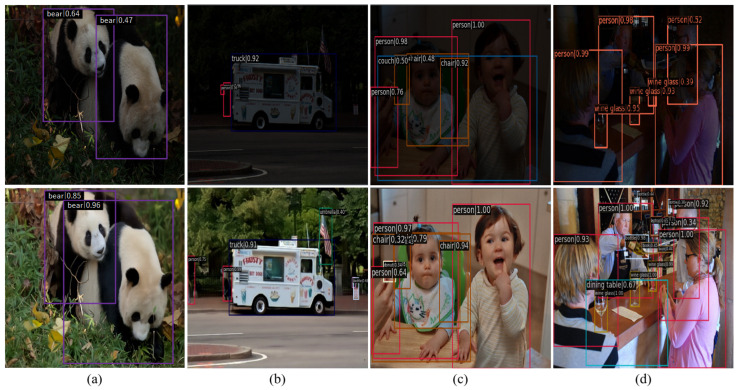
Comparison of object detection under low-light conditions. (**a**) shows the detection results of two giant pandas, (**b**) shows the detection results of a small car, (**c**,**d**) show the detection results of humans. The red and purple boxes in the figure highlight key areas of object detection to facilitate comparison of detection performance. From top to bottom are object detection results of original and light-enhanced images, respectively.

We also regard low-light image enhancement approaches as a pre-processing step for object detection under low-illumination conditions. Specifically, we first test several LLIE methods on the ExDark dataset [59], which comprises 5891 training images and 1472 test images taken under suboptimal lighting conditions, and further employ YOLO V5 on original and light-enhanced images to verify the effectiveness of the methods. Figure 12 demonstrates the object detection results of YOLO V5, and their corresponding average precision (AP) scores are shown in Table 9. It can be easily found that the LLIE methods can improve the performance of YOLO V5 under low-light conditions, and our LSUNet has superior performance in object detection in the dark compared with the other state-of-the-art LLIE methods.

## 5. Conclusions

In this paper, we propose a network called large–small-scale structure blended U-Net (LSUNet) for low-light image enhancement which includes color space transformation, a light-boosting network (LB-Net), and a color correction network (CC-Net). This method processes low-light images in the LAB color space to extract the luminosity and color components. LB-Net is built upon the enhanced U-shaped network to explore the hierarchical features of the luminosity component, and CC-Net relies on the large–small-scale structure to extract the multiscale features of the color component. Additionally, an efficient feature interaction module is introduced for the interaction of the two branches. Extensive experiments demonstrate that this method can be effectively used for low-light image enhancement and can further improve object detection under inadequate lighting conditions. Although our method shows better performance in light enhancement and color correction, it fails to remove local over-enhancement and inherent noise. In the future, we can integrate a brightness perception module and hand-craft priors in the designed LSUNet for addressing these challenges.

## Figures and Tables

**Table 1:** The parameters and details of the CC-Net model.

Input	Operator	Kernel Size	Channels	Output
Input_AB	ReLU(Conv)	3 × 3	32	Conv1_0
Conv1_0	ReLU(Conv)	3 × 3	64	Conv1_1
Conv1_1	ReLU(Conv)	1 × 1	128	Conv1_2
Conv1_2	ReLU(Conv)	3 × 3	64	Conv1_3
Conv1_3	Conv	3 × 3	2	Conv1_4
Conv1_4, Input_AB	⨂	-	2	Conv1
Input_AB	ReLU(Conv)	3 × 3	32	Conv2_0
Conv2_0	ReLU(Conv)	3 × 3	64	Conv2_1
Conv2_1	ReLU(Conv)	1 × 1	128	Conv2_2
Conv2_2	ReLU(Conv)	3 × 3	64	Conv2_3
Conv2_3	ReLU(Conv)	3 × 3	64	Conv2_4
Conv2_4	ReLU(Conv)	1 × 1	128	Conv2_5
Conv2_5	ReLU(Conv)	3 × 3	64	Conv2_6
Conv2_6	Conv	3 × 3	2	Conv2_7
Conv2_7, Input_AB	⨂	-	2	Conv2
Conv1, Conv2	⨁	-	2	Enhanced_AB

**Table 2:** Average AG, PSNR, and SSIM scores of MIT Adobe FiveK in different color spaces.

Color Spaces	PSNR ↑	SSIM ↑	AG ↑
RGB	22.57	0.8873	6.3470
HSV	22.91	0.8977	6.3598
YCbCr	22.03	0.8903	6.5210
Eigencolor	21.69	0.8867	6.5001
LAB	**23.62**	**0.9009**	**6.5378**

*Note: *↑ indicates that a higher value corresponds to better performance. The bold values indicate the performance results of our experiment in each column.

**Table 3:** Comparison of different modules in CC-Net on PSNR and SSIM.

Model	PSNR ↑	SSIM ↑
- w/o SM	21.73	0.8968
-w/o LM	22.99	0.8975
Ours	**23.62**	**0.9009**

*Note: *↑ indicates the higher the value, the better performance. The bold text indicates the best performance.

**Table 4:** The average PSNR and SSIM scores generated by different versions of LSUNet.

Loss Function	PSNR	SSIM
1. w/o $L_{1}$, w/o $L_{spa}$, w/o $L_{SSIM}$	17.03	0.6949
2. with $L_{1}$, w/o $L_{spa}$, w/o $L_{SSIM}$	18.44	0.7032
3. w/o $L_{1}$, with $L_{spa}$, w/o $L_{SSIM}$	18.41	0.7511
4. w/o $L_{1}$, w/o $L_{spa}$, with $L_{SSIM}$	18.43	0.8540
5. with $L_{1}$, with $L_{spa}$, w/o $L_{SSIM}$	18.55	0.8374
6. with $L_{1}$, w/o $L_{spa}$, with $L_{SSIM}$	19.84	0.8557
7. w/o $L_{1}$, with $L_{spa}$, with $L_{SSIM}$	19.72	0.8938

**Table 5:** Quantitative comparison of different methods on MIT-Adobe FiveK and LOL datasets.

	MIT-Adobe FiveK			LOL		
Method	PSNR ↑	SSIM ↑	LPIPS ↓	PSNR ↑	SSIM ↑	LPIPS ↓
RetinexNet [35]	18.31	0.7808	0.244	16.77	0.6613	0.280
EnlightenGAN [14]	19.49	0.8473	0.167	17.32	0.6785	0.215
KinD [36]	18.15	0.8024	0.121	18.21	0.7490	0.147
RRDNet [37]	15.31	0.7945	0.245	15.57	0.7029	0.229
Zero-DCE [41]	16.10	0.8359	0.203	14.58	0.6107	0.163
R2RNet [38]	19.00	0.8399	0.116	20.21	0.8460	0.085
LightenDiffusion [50]	19.96	0.8013	0.127	20.45	0.8035	0.192
CSPN [39]	23.57	**0.9291**	0.095	**23.82**	0.8543	0.085
DSLR [40]	18.58	0.5971	0.143	21.17	0.6921	0.201
Ours	**23.62**	0.9009	**0.039**	23.43	**0.8664**	**0.036**

*Note: *↑ indicates that a higher value represents better performance, and ↓ indicates that a lower value represents better performance. The bold values indicate the performance results of our experiment in each column.

**Table 6:** Quantitative comparison of different methods on MEF, Fusion, and VV datasets.

	MEF				Fusion				VV			
Method	NIQE ↓	NFERM ↓	IE ↑	LOE ↓	NIQE ↓	NFERM ↓	IE ↑	LOE ↓	NIQE ↓	NFERM ↓	IE ↑	LOE ↓
RetinexNet [35]	4.128	22.584	6.321	1258.403	4.069	15.507	7.098	1550.720	3.692	19.113	6.441	911.311
EnlightenGAN [14]	4.319	15.670	9.891	1567.019	3.972	13.391	8.652	1339.109	3.429	16.098	8.910	609.801
KinD [36]	3.718	18.758	7.252	875.813	4.1952	19.350	9.627	935.044	3.084	17.444	8.862	744.395
RRDNet [37]	3.605	20.508	6.907	605.083	3.458	9.041	7.573	904.111	2.625	15.456	7.126	544.622
Zero-DCE [41]	3.448	13.890	7.105	1389.023	3.820	11.346	7.943	1134.603	2.767	4.894	7.953	**489.423**
R2RNet [38]	4.195	**9.341**	7.647	934.145	3.809	9.694	9.036	969.601	3.356	6.528	8.121	652.811
CIDNet [51]	3.561	11.701	7.376	743.193	3.834	12.371	8.739	1203.746	2.876	4.972	7.803	598.367
LightenDiffusion [50]	3.992	12.976	7.112	799.840	4.001	12.574	8.652	1467.101	3.610	5.079	7.521	666.096
DSLR [40]	4.105	13.027	6.976	1199.605	3.998	15.599	6.987	1501.121	3.666	19.187	6.392	923.653
Ours	**3.382**	10.473	**14.087**	**404.736**	**3.050**	**7.541**	**12.528**	**754.142**	**2.577**	**5.345**	**11.824**	534.580

*Note: *↑ indicates that a higher value represents better performance, and ↓ indicates that a lower value represents better performance. The bold values indicate the performance results of our experiment in each column.

**Table 7:** Visual ratings of performance of different methods on MEF, Fusion, and VV datasets given by participants.

Method	MEF	Fusion	VV
RetinexNet [35]	1.3284	1.2776	1.3489
EnlightenGAN [14]	2.1698	3.9870	3.0982
KinD [36]	3.2476	3.4276	4.0128
RRDNet [37]	2.9488	3.6610	3.5821
Zero-DCE [41]	4.1852	3.5223	3.1326
R2RNet [38]	3.1689	2.1095	2.7691
CIDNet [51]	3.8897	3.9853	3.8985
LightenDiffusion [50]	3.4453	3.8769	3.7741
DSLR [40]	3.0698	3.6672	2.9978
Ours	**4.3372**	**4.2769**	**4.2010**

*Note:* The bold values indicate the performance results of our experiment in each column.

**Table 8:** Computational complexity comparison of existing LLIE methods on LOL benchmark.

Method	Param (M) ↓	Flops (G) ↓	Time (s) ↓
RetinexNet [35]	1.23	6.79	0.5217
KinD [36]	8.49	7.44	0.6445
RRDNet [37]	28.83	53.47	0.9763
Zero-DCE [41]	1.21	5.21	0.007
R2RNet [38]	-	-	0.6894
CIDNet [51]	1.98	8.03	0.7869
LightenDiffusion [50]	101.71	210	1.2001
EnlightenGAN [14]	8.64	7.88	0.6501
CSPN [39]	60.921	1.40	0.149
DSLR [40]	14.31	22.95	0.9210
Ours	**1.01**	**4.13**	**0.0059**

*Note:*↓ indicates that a lower value represents better performance. The bold values indicate the performance results of our experiment in each column.

**Table 9:** Quantitative analysis of our method on original/enhanced ExDark.

Input	Bicycle	Boat	Bottle	Bus	Car	Cat	Chair	Cup	Dog	Motor	People	Table
Original	70.3	61.3	54.7	70.3	61.3	50.1	35.9	46.4	51.7	51.3	46.8	34.3
Enhanced	76.1	66.0	65.7	82.9	81.9	60.1	59.1	68.8	62.3	65.0	65.9	48.1

## Data Availability

The data supporting the findings of this study are publicly available. No new data were created in this study.
